# (2,3,7,8,12,13,17,18-Octa­ethyl­por­phin­ato)(trifluoro­methane­sulfonato)iron(III)

**DOI:** 10.1107/S1600536808031504

**Published:** 2008-10-04

**Authors:** Nan Xu, Douglas R. Powell, George B. Richter-Addo

**Affiliations:** a Department of Chemistry and Biochemistry, University of Oklahoma, 620 Parrington Oval, Norman, OK 73019, USA

## Abstract

The title compound, [Fe(CF_3_O_3_S)(C_36_H_44_N_4_)], is an iron(III) porphyrin complex with the trifluoro­methane­sulfonate anion as an axial ligand. The Fe atom is displaced by 0.219 (2) Å toward the trifluoro­methane­sulfonate anion from the 24-atom mean plane of the porphyrin, resulting in a distorted FeN_4_O square-based pyramidal geometry. One ethyl­ene group is disordered over two orientations in a 0.502 (6):0.498 (6) ratio.

## Related literature

For the structures of other related porphyrin (‘picket-fence’, tetra­phenyl­porphyrin) derivatives, see: González & Wilson (1994 ▶); Gismelseed *et al.* (1990 ▶).
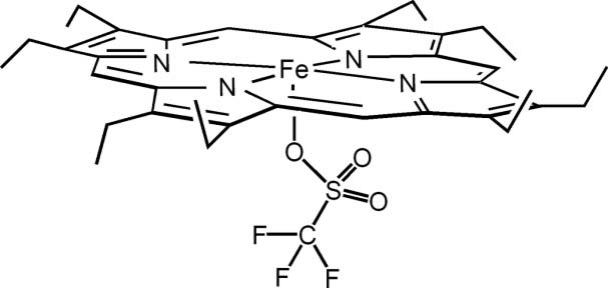


## Experimental

### 

#### Crystal data

[Fe(CF_3_O_3_S)(C_36_H_44_N_4_)]
*M* *_r_* = 737.67
Triclinic, 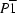

*a* = 12.2180 (14) Å
*b* = 12.7994 (15) Å
*c* = 13.8028 (16) Å
α = 96.324 (5)°
β = 115.007 (5)°
γ = 111.721 (6)°
*V* = 1723.8 (4) Å^3^
*Z* = 2
Mo *K*α radiation
μ = 0.56 mm^−1^
*T* = 100 (2) K
0.52 × 0.36 × 0.35 mm

#### Data collection

Bruker APEX CCD diffractometer
Absorption correction: multi-scan (*SADABS*; Sheldrick, 2007 ▶) *T*_min_ = 0.760, *T*_max_ = 0.829
17956 measured reflections
6731 independent reflections
6207 reflections with *I* > 2σ(*I*)
*R*_int_ = 0.019

#### Refinement

*R*[*F* ^2^ > 2σ(*F* ^2^)] = 0.038
*wR*(*F* ^2^) = 0.107
*S* = 1.04
6731 reflections
452 parameters
26 restraints
H-atom parameters constrained
Δρ_max_ = 1.02 e Å^−3^
Δρ_min_ = −0.65 e Å^−3^

### 

Data collection: *SMART* (Bruker, 1998 ▶); cell refinement: *SAINT* (Bruker, 1998 ▶); data reduction: *SAINT*; program(s) used to solve structure: *SHELXTL* (Sheldrick, 2008 ▶); program(s) used to refine structure: *SHELXTL*; molecular graphics: *SHELXTL*; software used to prepare material for publication: *SHELXTL*.

## Supplementary Material

Crystal structure: contains datablocks I, global. DOI: 10.1107/S1600536808031504/hb2808sup1.cif

Structure factors: contains datablocks I. DOI: 10.1107/S1600536808031504/hb2808Isup2.hkl

Additional supplementary materials:  crystallographic information; 3D view; checkCIF report

## Figures and Tables

**Table:** 

Fe1—N1	1.9979 (17)
Fe1—N2	1.9981 (17)
Fe1—N3	1.9999 (16)
Fe1—N4	2.0001 (17)
Fe1—O1*A*	2.0392 (14)

**Table:** 

S1*A*—O1*A*—Fe1	129.34 (8)

**Fig. 1.**
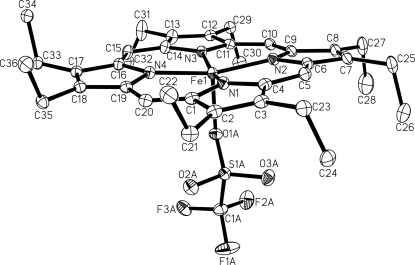
The molecular structure of (I) with displacement ellipsoids drawn at the 35% probability level. H atoms and the minor disorder component are omitted for clarity.

## supplementary crystallographic information

### Comment

Many iron porphyrin complexes have been synthesized as models for the study of the important roles that heme enzymes play in biological processes. In this paper, we report the first structure of the title compound, (I), five-coordinate (trifluoromethanesulfonato)(octaethylporphinato)iron(III). Other trifluoromethanesulfonato iron porphyrin derivatives have been reported previously: The (T_piv_PP)Fe(OSO_2_CF_3_)(H_2_O) compound is six-coordinate at Fe, and the (TPP)Fe(OSO_2_CF_3_) is five coordinate at Fe (González & Wilson 1994 and Gismelseed *et al.* 1990).

The molecular structure of (I) is shown in Fig. 1. The porphyrin core of the compound is moderately ruffled. The iron atom is displaced by 0.219 (2) Å from the 24-atom mean porphyrin plane toward the trifluoromethanesulfonate anion. The trifluoromethanesulfonate anion binds to the iron center through one of its sulfonato oxygen atoms. The Fe—O distance of 2.0392 (14) Å is longer than those of the five-coordinate tetraphenylporphyrin derivative [1.946 (6) Å - 2.022 (3) Å] (González & Wilson 1994) and the six-coordinate picket-fence porphyrin derivative [2.188 (5) Å) (Gismelseed *et al.*,  1990). The Fe—N_p_ distances are 1.9979 (17) Å - 2.0001 (17) Å (Table 1). The bond angle of the Fe—O—S linkage is 129.34 (8) °.

### Experimental

To a toluene solution (20 ml) of (octaethylporphinato)FeCl (0.015 g, 0.024 mmol) (purchased from Mid-Century Chemical Inc.) under N_2_ was added silver trifluoromethanesulfonate (0.0068 g, 0.026 mmol) (purchased from Aldrich Chemical Company and used as received). The resulting mixture was stirred for 2 h and filtered into a clean Schlenk tube under N_2_. A red powder was obtained after removal of the solvent under vacuum. A suitable black prism of (I) was grown by slow evaporation of a dichloromethane-hexane (1:1 v/v) solution of the complex at room temperature under N_2_.

### Refinement

H atoms were positioned geometrically and refined using a riding model with C—H = 0.95 Å for aromatic carbons, 0.98 Å for methylene carbons and 0.99 Å for methyl carbons. One ethylene group (C31—C32) is disordered and is modeled in two orientations, with occupancies refined to 0.502 (6) and 0.498 (6) for the unprimed and primed atoms, respectively.

### Crystal data

**Table:**

[Fe(CF_3_O_3_S)(C_36_H_44_N_4_)]	*Z* = 2
*M**_r_* = 737.67	*F*(000) = 774
Triclinic, *P*1	*D*_x_ = 1.421 Mg m^−^^3^
Hall symbol: -P 1	Mo *K*α radiation, λ = 0.71073 Å
*a* = 12.2180 (14) Å	Cell parameters from 7756 reflections
*b* = 12.7994 (15) Å	θ = 2.3–28.3°
*c* = 13.8028 (16) Å	µ = 0.56 mm^−^^1^
α = 96.324 (5)°	*T* = 100 K
β = 115.007 (5)°	Prism, black
γ = 111.721 (6)°	0.52 × 0.36 × 0.35 mm
*V* = 1723.8 (4) Å^3^	

### Data collection

**Table:**

Bruker APEX CCD diffractometer	6731 independent reflections
Radiation source: fine-focus sealed tube	6207 reflections with *I* > 2σ(*I*)
graphite	*R*_int_ = 0.019
ω scans	θ_max_ = 26.0°, θ_min_ = 1.7°
Absorption correction: multi-scan (SADABS; Sheldrick, 2007)	*h* = −15→15
*T*_min_ = 0.760, *T*_max_ = 0.829	*k* = −15→15
17956 measured reflections	*l* = −17→17

### Refinement

**Table:**

Refinement on *F*^2^	Primary atom site location: structure-invariant direct methods
Least-squares matrix: full	Secondary atom site location: difference Fourier map
*R*[*F*^2^ > 2σ(*F*^2^)] = 0.038	Hydrogen site location: inferred from neighbouring sites
*wR*(*F*^2^) = 0.107	H-atom parameters constrained
*S* = 1.04	*w* = 1/[σ^2^(*F*_o_^2^) + (0.058*P*)^2^ + 1.5*P*] where *P* = (*F*_o_^2^ + 2*F*_c_^2^)/3
6731 reflections	(Δ/σ)_max_ = 0.001
452 parameters	Δρ_max_ = 1.02 e Å^−^^3^
26 restraints	Δρ_min_ = −0.65 e Å^−^^3^

### Special details

**Table:**

Geometry. All e.s.d.'s (except the e.s.d. in the dihedral angle between two l.s. planes) are estimated using the full covariance matrix. The cell e.s.d.'s are taken into account individually in the estimation of e.s.d.'s in distances, angles and torsion angles; correlations between e.s.d.'s in cell parameters are only used when they are defined by crystal symmetry. An approximate (isotropic) treatment of cell e.s.d.'s is used for estimating e.s.d.'s involving l.s. planes.
Refinement. Restraints on the positional and displacement parameters of the disordered atoms were required.

### Fractional atomic coordinates and isotropic or equivalent isotropic displacement parameters (Å^2^)

**Table:**

	*x*	*y*	*z*	*U*_iso_*/*U*_eq_	Occ. (<1)
Fe1	0.41603 (3)	0.34987 (2)	0.31778 (2)	0.01775 (10)	
N1	0.44180 (17)	0.27844 (15)	0.44231 (13)	0.0200 (3)	
N2	0.59488 (17)	0.49255 (14)	0.42231 (13)	0.0204 (3)	
N3	0.37474 (16)	0.43983 (14)	0.20913 (13)	0.0189 (3)	
N4	0.22023 (16)	0.22759 (14)	0.23117 (13)	0.0192 (3)	
C1	0.3569 (2)	0.16671 (18)	0.43492 (16)	0.0205 (4)	
C2	0.4213 (2)	0.13718 (19)	0.53472 (17)	0.0224 (4)	
C3	0.5444 (2)	0.23325 (19)	0.60530 (16)	0.0226 (4)	
C4	0.5575 (2)	0.32002 (18)	0.54697 (16)	0.0214 (4)	
C5	0.6719 (2)	0.42714 (19)	0.58778 (16)	0.0229 (4)	
H5	0.7433	0.4485	0.6626	0.028*	
C6	0.6912 (2)	0.50594 (18)	0.52876 (16)	0.0223 (4)	
C7	0.8168 (2)	0.61118 (18)	0.56848 (17)	0.0259 (4)	
C8	0.7974 (2)	0.66287 (18)	0.48569 (18)	0.0268 (4)	
C9	0.6592 (2)	0.58940 (17)	0.39561 (17)	0.0223 (4)	
C10	0.5988 (2)	0.61321 (17)	0.29676 (17)	0.0223 (4)	
H10	0.6531	0.6825	0.2876	0.027*	
C11	0.4660 (2)	0.54473 (17)	0.21018 (16)	0.0193 (4)	
C12	0.4026 (2)	0.57402 (18)	0.11033 (17)	0.0219 (4)	
C13	0.2715 (2)	0.4872 (2)	0.04836 (18)	0.0270 (4)	
C14	0.2548 (2)	0.40375 (18)	0.10955 (17)	0.0224 (4)	
C15	0.1357 (2)	0.30184 (19)	0.07331 (17)	0.0239 (4)	
H15	0.0588	0.2873	0.0041	0.029*	
C16	0.1192 (2)	0.21942 (18)	0.12914 (16)	0.0203 (4)	
C17	−0.0046 (2)	0.11087 (18)	0.08721 (16)	0.0202 (4)	
C18	0.0227 (2)	0.05058 (17)	0.16272 (16)	0.0205 (4)	
C19	0.1613 (2)	0.12371 (17)	0.25172 (16)	0.0197 (4)	
C20	0.2268 (2)	0.09456 (18)	0.34568 (17)	0.0210 (4)	
H20	0.1784	0.0187	0.3491	0.025*	
C21	0.3620 (2)	0.02166 (19)	0.55482 (18)	0.0261 (4)	
H21A	0.4372	0.0066	0.6046	0.031*	
H21B	0.3057	−0.0431	0.4820	0.031*	
C22	0.2749 (2)	0.0183 (2)	0.6080 (2)	0.0328 (5)	
H22A	0.3298	0.0818	0.6804	0.049*	
H22B	0.2410	−0.0585	0.6201	0.049*	
H22C	0.1977	0.0295	0.5578	0.049*	
C23	0.6511 (2)	0.2489 (2)	0.72072 (17)	0.0266 (4)	
H23A	0.6057	0.2023	0.7574	0.032*	
H23B	0.6997	0.3335	0.7666	0.032*	
C24	0.7551 (2)	0.2103 (2)	0.71934 (19)	0.0307 (5)	
H24A	0.7098	0.1243	0.6834	0.046*	
H24B	0.8276	0.2312	0.7968	0.046*	
H24C	0.7945	0.2506	0.6770	0.046*	
C25	0.9460 (2)	0.6459 (2)	0.67638 (19)	0.0323 (5)	
H25A	0.9242	0.6299	0.7363	0.039*	
H25B	1.0062	0.7320	0.6999	0.039*	
C26	1.0208 (2)	0.5775 (2)	0.6629 (2)	0.0402 (6)	
H26A	0.9593	0.4921	0.6345	0.060*	
H26B	1.1001	0.5968	0.7360	0.060*	
H26C	1.0510	0.5996	0.6093	0.060*	
C27	0.8991 (2)	0.7701 (2)	0.4815 (2)	0.0386 (6)	
H27A	0.9722	0.8206	0.5594	0.046*	
H27B	0.8536	0.8166	0.4463	0.046*	
C28	0.9625 (3)	0.7402 (3)	0.4163 (3)	0.0550 (8)	
H28A	1.0171	0.7027	0.4557	0.083*	
H28B	1.0210	0.8130	0.4102	0.083*	
H28C	0.8904	0.6856	0.3407	0.083*	
C29	0.4719 (2)	0.68032 (18)	0.08217 (18)	0.0238 (4)	
H29A	0.5382	0.7480	0.1525	0.029*	
H29B	0.4030	0.7021	0.0330	0.029*	
C30	0.5464 (2)	0.6586 (2)	0.0229 (2)	0.0305 (5)	
H30A	0.6139	0.6360	0.0707	0.046*	
H30B	0.5923	0.7313	0.0090	0.046*	
H30C	0.4805	0.5948	−0.0491	0.046*	
C31	0.1635 (2)	0.4765 (2)	−0.06200 (19)	0.0410 (6)	
H31A	0.0760	0.4415	−0.0626	0.049*	0.502 (6)
H31B	0.1824	0.5577	−0.0657	0.049*	0.502 (6)
H31C	0.1218	0.3954	−0.1133	0.049*	0.498 (6)
H31D	0.2079	0.5320	−0.0939	0.049*	0.498 (6)
C32	0.1439 (5)	0.4092 (4)	−0.1630 (3)	0.0315 (12)	0.502 (6)
H32A	0.2232	0.4498	−0.1715	0.047*	0.502 (6)
H32B	0.0621	0.4013	−0.2279	0.047*	0.502 (6)
H32C	0.1330	0.3303	−0.1588	0.047*	0.502 (6)
C32'	0.0532 (4)	0.4962 (4)	−0.0675 (4)	0.0309 (12)	0.498 (6)
H32D	−0.0091	0.4857	−0.1457	0.046*	0.498 (6)
H32E	0.0893	0.5770	−0.0203	0.046*	0.498 (6)
H32F	0.0040	0.4393	−0.0403	0.046*	0.498 (6)
C33	−0.1365 (2)	0.07843 (18)	−0.01692 (17)	0.0218 (4)	
H33A	−0.1968	−0.0077	−0.0408	0.026*	
H33B	−0.1182	0.0952	−0.0784	0.026*	
C34	−0.2093 (2)	0.1466 (2)	0.00162 (19)	0.0288 (5)	
H34A	−0.2287	0.1295	0.0617	0.043*	
H34B	−0.2949	0.1223	−0.0682	0.043*	
H34C	−0.1510	0.2319	0.0233	0.043*	
C35	−0.0705 (2)	−0.06856 (18)	0.15773 (18)	0.0238 (4)	
H35A	−0.0177	−0.1122	0.1854	0.029*	
H35B	−0.1420	−0.1144	0.0782	0.029*	
C36	−0.1385 (2)	−0.0610 (2)	0.2272 (2)	0.0322 (5)	
H36A	−0.0684	−0.0145	0.3058	0.048*	
H36B	−0.1947	−0.1411	0.2233	0.048*	
H36C	−0.1960	−0.0227	0.1971	0.048*	
S1A	0.53812 (5)	0.18176 (4)	0.27122 (4)	0.02140 (13)	
O1A	0.48331 (14)	0.26554 (12)	0.23952 (11)	0.0226 (3)	
O2A	0.43782 (16)	0.06943 (13)	0.25787 (13)	0.0294 (3)	
O3A	0.66892 (16)	0.23466 (15)	0.37091 (13)	0.0323 (4)	
C1A	0.5698 (2)	0.1493 (2)	0.15635 (19)	0.0280 (5)	
F1A	0.6203 (2)	0.07312 (17)	0.16842 (14)	0.0545 (5)	
F2A	0.65782 (14)	0.24699 (14)	0.15333 (12)	0.0415 (4)	
F3A	0.45686 (14)	0.10225 (12)	0.05638 (10)	0.0338 (3)	

### Atomic displacement parameters (Å^2^)

**Table:**

	*U*^11^	*U*^22^	*U*^33^	*U*^12^	*U*^13^	*U*^23^
Fe1	0.01791 (16)	0.01937 (16)	0.01339 (15)	0.00727 (12)	0.00684 (12)	0.00551 (11)
N1	0.0201 (8)	0.0239 (8)	0.0152 (8)	0.0098 (7)	0.0086 (7)	0.0058 (7)
N2	0.0217 (8)	0.0208 (8)	0.0151 (8)	0.0093 (7)	0.0072 (7)	0.0044 (6)
N3	0.0185 (8)	0.0204 (8)	0.0160 (8)	0.0079 (7)	0.0082 (6)	0.0054 (6)
N4	0.0201 (8)	0.0222 (8)	0.0160 (8)	0.0092 (7)	0.0098 (7)	0.0074 (6)
C1	0.0255 (10)	0.0251 (10)	0.0185 (9)	0.0142 (8)	0.0144 (8)	0.0097 (8)
C2	0.0281 (10)	0.0291 (11)	0.0189 (9)	0.0173 (9)	0.0148 (8)	0.0111 (8)
C3	0.0279 (10)	0.0306 (11)	0.0167 (9)	0.0173 (9)	0.0134 (8)	0.0099 (8)
C4	0.0251 (10)	0.0285 (10)	0.0153 (9)	0.0154 (9)	0.0114 (8)	0.0077 (8)
C5	0.0244 (10)	0.0287 (11)	0.0130 (9)	0.0135 (9)	0.0067 (8)	0.0043 (8)
C6	0.0243 (10)	0.0235 (10)	0.0150 (9)	0.0115 (8)	0.0072 (8)	0.0018 (8)
C7	0.0265 (11)	0.0207 (10)	0.0190 (10)	0.0090 (8)	0.0052 (8)	−0.0001 (8)
C8	0.0251 (10)	0.0190 (10)	0.0234 (10)	0.0071 (8)	0.0055 (9)	0.0020 (8)
C9	0.0227 (10)	0.0186 (9)	0.0201 (10)	0.0084 (8)	0.0081 (8)	0.0030 (8)
C10	0.0240 (10)	0.0169 (9)	0.0231 (10)	0.0075 (8)	0.0112 (8)	0.0061 (8)
C11	0.0227 (10)	0.0182 (9)	0.0188 (9)	0.0100 (8)	0.0114 (8)	0.0056 (7)
C12	0.0245 (10)	0.0241 (10)	0.0209 (10)	0.0122 (8)	0.0132 (8)	0.0091 (8)
C13	0.0243 (10)	0.0314 (11)	0.0235 (10)	0.0104 (9)	0.0112 (9)	0.0151 (9)
C14	0.0207 (10)	0.0271 (10)	0.0189 (9)	0.0104 (8)	0.0094 (8)	0.0104 (8)
C15	0.0186 (9)	0.0305 (11)	0.0169 (9)	0.0090 (8)	0.0057 (8)	0.0100 (8)
C16	0.0188 (9)	0.0240 (10)	0.0177 (9)	0.0089 (8)	0.0098 (8)	0.0061 (8)
C17	0.0194 (9)	0.0236 (10)	0.0180 (9)	0.0090 (8)	0.0107 (8)	0.0053 (8)
C18	0.0210 (10)	0.0223 (10)	0.0188 (9)	0.0082 (8)	0.0124 (8)	0.0050 (8)
C19	0.0215 (9)	0.0222 (9)	0.0178 (9)	0.0097 (8)	0.0122 (8)	0.0060 (8)
C20	0.0248 (10)	0.0217 (9)	0.0211 (10)	0.0110 (8)	0.0146 (8)	0.0091 (8)
C21	0.0331 (11)	0.0296 (11)	0.0226 (10)	0.0177 (9)	0.0156 (9)	0.0135 (9)
C22	0.0366 (12)	0.0290 (11)	0.0352 (12)	0.0112 (10)	0.0236 (11)	0.0097 (10)
C23	0.0325 (11)	0.0338 (11)	0.0162 (10)	0.0180 (10)	0.0116 (9)	0.0104 (8)
C24	0.0302 (11)	0.0394 (13)	0.0224 (10)	0.0194 (10)	0.0099 (9)	0.0107 (9)
C25	0.0268 (11)	0.0248 (11)	0.0220 (11)	0.0045 (9)	0.0006 (9)	0.0027 (9)
C26	0.0268 (12)	0.0499 (15)	0.0345 (13)	0.0161 (11)	0.0085 (10)	0.0159 (11)
C27	0.0256 (11)	0.0225 (11)	0.0374 (13)	0.0014 (9)	−0.0008 (10)	0.0092 (10)
C28	0.0390 (15)	0.0484 (16)	0.0609 (19)	0.0043 (13)	0.0232 (14)	0.0250 (15)
C29	0.0262 (10)	0.0225 (10)	0.0231 (10)	0.0107 (8)	0.0123 (8)	0.0107 (8)
C30	0.0347 (12)	0.0292 (11)	0.0370 (12)	0.0151 (10)	0.0237 (10)	0.0177 (10)
C31	0.0256 (11)	0.0444 (14)	0.0335 (12)	0.0043 (10)	0.0054 (10)	0.0271 (11)
C32	0.032 (2)	0.036 (2)	0.022 (2)	0.017 (2)	0.0099 (18)	0.0087 (18)
C32'	0.027 (2)	0.032 (2)	0.033 (2)	0.0138 (19)	0.0125 (19)	0.0150 (19)
C33	0.0181 (9)	0.0234 (10)	0.0192 (9)	0.0071 (8)	0.0080 (8)	0.0051 (8)
C34	0.0228 (10)	0.0283 (11)	0.0281 (11)	0.0120 (9)	0.0081 (9)	0.0037 (9)
C35	0.0248 (10)	0.0207 (10)	0.0234 (10)	0.0070 (8)	0.0132 (8)	0.0060 (8)
C36	0.0341 (12)	0.0297 (11)	0.0364 (12)	0.0097 (10)	0.0249 (11)	0.0104 (10)
S1A	0.0230 (3)	0.0241 (3)	0.0178 (2)	0.0112 (2)	0.0105 (2)	0.00716 (19)
O1A	0.0287 (7)	0.0248 (7)	0.0194 (7)	0.0143 (6)	0.0143 (6)	0.0084 (6)
O2A	0.0342 (8)	0.0261 (8)	0.0283 (8)	0.0117 (7)	0.0171 (7)	0.0123 (6)
O3A	0.0264 (8)	0.0429 (9)	0.0215 (8)	0.0160 (7)	0.0075 (6)	0.0087 (7)
C1A	0.0326 (11)	0.0331 (12)	0.0265 (11)	0.0190 (10)	0.0178 (9)	0.0105 (9)
F1A	0.0885 (13)	0.0807 (12)	0.0476 (9)	0.0708 (11)	0.0473 (9)	0.0348 (9)
F2A	0.0325 (7)	0.0558 (9)	0.0351 (8)	0.0123 (7)	0.0227 (6)	0.0157 (7)
F3A	0.0429 (8)	0.0323 (7)	0.0198 (6)	0.0137 (6)	0.0146 (6)	0.0046 (5)

### Geometric parameters (Å, °)

**Table:**

Fe1—N1	1.9979 (17)	C23—H23B	0.9900
Fe1—N2	1.9981 (17)	C24—H24A	0.9800
Fe1—N3	1.9999 (16)	C24—H24B	0.9800
Fe1—N4	2.0001 (17)	C24—H24C	0.9800
Fe1—O1A	2.0392 (14)	C25—C26	1.529 (4)
N1—C1	1.383 (3)	C25—H25A	0.9900
N1—C4	1.384 (2)	C25—H25B	0.9900
N2—C6	1.381 (3)	C26—H26A	0.9800
N2—C9	1.383 (3)	C26—H26B	0.9800
N3—C14	1.381 (2)	C26—H26C	0.9800
N3—C11	1.386 (2)	C27—C28	1.512 (4)
N4—C19	1.383 (2)	C27—H27A	0.9900
N4—C16	1.386 (2)	C27—H27B	0.9900
C1—C20	1.380 (3)	C28—H28A	0.9800
C1—C2	1.442 (3)	C28—H28B	0.9800
C2—C3	1.361 (3)	C28—H28C	0.9800
C2—C21	1.502 (3)	C29—C30	1.529 (3)
C3—C4	1.443 (3)	C29—H29A	0.9900
C3—C23	1.502 (3)	C29—H29B	0.9900
C4—C5	1.377 (3)	C30—H30A	0.9800
C5—C6	1.380 (3)	C30—H30B	0.9800
C5—H5	0.9500	C30—H30C	0.9800
C6—C7	1.437 (3)	C31—C32	1.430 (3)
C7—C8	1.361 (3)	C31—C32'	1.433 (3)
C7—C25	1.502 (3)	C31—H31A	0.9900
C8—C9	1.440 (3)	C31—H31B	0.9900
C8—C27	1.500 (3)	C31—H31C	0.9900
C9—C10	1.381 (3)	C31—H31D	0.9900
C10—C11	1.380 (3)	C32—H32A	0.9800
C10—H10	0.9500	C32—H32B	0.9800
C11—C12	1.437 (3)	C32—H32C	0.9800
C12—C13	1.359 (3)	C32'—H32D	0.9800
C12—C29	1.504 (3)	C32'—H32E	0.9800
C13—C14	1.442 (3)	C32'—H32F	0.9800
C13—C31	1.484 (3)	C33—C34	1.529 (3)
C14—C15	1.380 (3)	C33—H33A	0.9900
C15—C16	1.379 (3)	C33—H33B	0.9900
C15—H15	0.9500	C34—H34A	0.9800
C16—C17	1.443 (3)	C34—H34B	0.9800
C17—C18	1.366 (3)	C34—H34C	0.9800
C17—C33	1.500 (3)	C35—C36	1.527 (3)
C18—C19	1.438 (3)	C35—H35A	0.9900
C18—C35	1.501 (3)	C35—H35B	0.9900
C19—C20	1.383 (3)	C36—H36A	0.9800
C20—H20	0.9500	C36—H36B	0.9800
C21—C22	1.519 (3)	C36—H36C	0.9800
C21—H21A	0.9900	S1A—O3A	1.4284 (16)
C21—H21B	0.9900	S1A—O2A	1.4316 (16)
C22—H22A	0.9800	S1A—O1A	1.4755 (15)
C22—H22B	0.9800	S1A—C1A	1.825 (2)
C22—H22C	0.9800	C1A—F1A	1.324 (3)
C23—C24	1.531 (3)	C1A—F3A	1.328 (3)
C23—H23A	0.9900	C1A—F2A	1.333 (3)
			
N1—Fe1—N2	89.36 (7)	H24A—C24—H24B	109.5
N1—Fe1—N3	167.25 (7)	C23—C24—H24C	109.5
N2—Fe1—N3	89.17 (7)	H24A—C24—H24C	109.5
N1—Fe1—N4	89.01 (7)	H24B—C24—H24C	109.5
N2—Fe1—N4	165.89 (7)	C7—C25—C26	111.46 (19)
N3—Fe1—N4	89.34 (7)	C7—C25—H25A	109.3
N1—Fe1—O1A	97.97 (6)	C26—C25—H25A	109.3
N2—Fe1—O1A	97.48 (6)	C7—C25—H25B	109.3
N3—Fe1—O1A	94.78 (6)	C26—C25—H25B	109.3
N4—Fe1—O1A	96.63 (6)	H25A—C25—H25B	108.0
C1—N1—C4	104.98 (16)	C25—C26—H26A	109.5
C1—N1—Fe1	126.65 (13)	C25—C26—H26B	109.5
C4—N1—Fe1	127.49 (14)	H26A—C26—H26B	109.5
C6—N2—C9	104.94 (16)	C25—C26—H26C	109.5
C6—N2—Fe1	127.33 (14)	H26A—C26—H26C	109.5
C9—N2—Fe1	126.74 (13)	H26B—C26—H26C	109.5
C14—N3—C11	104.97 (16)	C8—C27—C28	113.3 (2)
C14—N3—Fe1	127.55 (13)	C8—C27—H27A	108.9
C11—N3—Fe1	126.99 (13)	C28—C27—H27A	108.9
C19—N4—C16	104.82 (16)	C8—C27—H27B	108.9
C19—N4—Fe1	127.04 (13)	C28—C27—H27B	108.9
C16—N4—Fe1	127.53 (13)	H27A—C27—H27B	107.7
C20—C1—N1	124.70 (18)	C27—C28—H28A	109.5
C20—C1—C2	124.55 (19)	C27—C28—H28B	109.5
N1—C1—C2	110.73 (18)	H28A—C28—H28B	109.5
C3—C2—C1	106.82 (18)	C27—C28—H28C	109.5
C3—C2—C21	127.42 (19)	H28A—C28—H28C	109.5
C1—C2—C21	125.76 (19)	H28B—C28—H28C	109.5
C2—C3—C4	106.78 (17)	C12—C29—C30	112.86 (17)
C2—C3—C23	128.31 (19)	C12—C29—H29A	109.0
C4—C3—C23	124.89 (19)	C30—C29—H29A	109.0
C5—C4—N1	124.71 (18)	C12—C29—H29B	109.0
C5—C4—C3	124.58 (19)	C30—C29—H29B	109.0
N1—C4—C3	110.65 (18)	H29A—C29—H29B	107.8
C4—C5—C6	125.30 (19)	C29—C30—H30A	109.5
C4—C5—H5	117.4	C29—C30—H30B	109.5
C6—C5—H5	117.4	H30A—C30—H30B	109.5
C5—C6—N2	124.88 (19)	C29—C30—H30C	109.5
C5—C6—C7	124.30 (19)	H30A—C30—H30C	109.5
N2—C6—C7	110.78 (18)	H30B—C30—H30C	109.5
C8—C7—C6	106.90 (18)	C32—C31—C13	118.9 (2)
C8—C7—C25	128.4 (2)	C32'—C31—C13	119.5 (2)
C6—C7—C25	124.4 (2)	C32—C31—H31A	107.6
C7—C8—C9	106.67 (19)	C13—C31—H31A	107.6
C7—C8—C27	128.4 (2)	C32—C31—H31B	107.6
C9—C8—C27	124.8 (2)	C13—C31—H31B	107.6
C10—C9—N2	124.71 (18)	H31A—C31—H31B	107.0
C10—C9—C8	124.58 (19)	C32'—C31—H31C	106.4
N2—C9—C8	110.71 (18)	C13—C31—H31C	107.9
C11—C10—C9	125.40 (19)	C32'—C31—H31D	107.7
C11—C10—H10	117.3	C13—C31—H31D	107.7
C9—C10—H10	117.3	H31C—C31—H31D	107.0
C10—C11—N3	124.51 (18)	H31C—C32—H31D	82.8
C10—C11—C12	124.67 (18)	C31—C32—H32A	109.5
N3—C11—C12	110.80 (17)	C31—C32—H32B	109.5
C13—C12—C11	106.66 (18)	H32A—C32—H32B	109.5
C13—C12—C29	127.99 (19)	C31—C32—H32C	109.5
C11—C12—C29	125.35 (18)	H32A—C32—H32C	109.5
C12—C13—C14	107.08 (18)	H32B—C32—H32C	109.5
C12—C13—C31	127.9 (2)	C31—C32'—H32D	109.5
C14—C13—C31	125.07 (19)	C31—C32'—H32E	109.5
C15—C14—N3	124.81 (18)	H32D—C32'—H32E	109.5
C15—C14—C13	124.70 (19)	C31—C32'—H32F	109.5
N3—C14—C13	110.49 (17)	H32D—C32'—H32F	109.5
C16—C15—C14	125.43 (19)	H32E—C32'—H32F	109.5
C16—C15—H15	117.3	C17—C33—C34	112.30 (17)
C14—C15—H15	117.3	C17—C33—H33A	109.1
C15—C16—N4	124.71 (18)	C34—C33—H33A	109.1
C15—C16—C17	124.46 (18)	C17—C33—H33B	109.1
N4—C16—C17	110.80 (17)	C34—C33—H33B	109.1
C18—C17—C16	106.55 (17)	H33A—C33—H33B	107.9
C18—C17—C33	128.90 (18)	C33—C34—H34A	109.5
C16—C17—C33	124.49 (18)	C33—C34—H34B	109.5
C17—C18—C19	106.85 (17)	H34A—C34—H34B	109.5
C17—C18—C35	128.19 (18)	C33—C34—H34C	109.5
C19—C18—C35	124.96 (18)	H34A—C34—H34C	109.5
C20—C19—N4	124.46 (18)	H34B—C34—H34C	109.5
C20—C19—C18	124.60 (19)	C18—C35—C36	113.27 (17)
N4—C19—C18	110.94 (17)	C18—C35—H35A	108.9
C1—C20—C19	125.27 (19)	C36—C35—H35A	108.9
C1—C20—H20	117.4	C18—C35—H35B	108.9
C19—C20—H20	117.4	C36—C35—H35B	108.9
C2—C21—C22	113.67 (18)	H35A—C35—H35B	107.7
C2—C21—H21A	108.8	C35—C36—H36A	109.5
C22—C21—H21A	108.8	C35—C36—H36B	109.5
C2—C21—H21B	108.8	H36A—C36—H36B	109.5
C22—C21—H21B	108.8	C35—C36—H36C	109.5
H21A—C21—H21B	107.7	H36A—C36—H36C	109.5
C21—C22—H22A	109.5	H36B—C36—H36C	109.5
C21—C22—H22B	109.5	O3A—S1A—O2A	117.97 (10)
H22A—C22—H22B	109.5	O3A—S1A—O1A	114.00 (9)
C21—C22—H22C	109.5	O2A—S1A—O1A	113.34 (9)
H22A—C22—H22C	109.5	O3A—S1A—C1A	104.86 (10)
H22B—C22—H22C	109.5	O2A—S1A—C1A	103.69 (10)
C3—C23—C24	113.37 (17)	O1A—S1A—C1A	100.22 (9)
C3—C23—H23A	108.9	S1A—O1A—Fe1	129.34 (8)
C24—C23—H23A	108.9	F1A—C1A—F3A	107.70 (18)
C3—C23—H23B	108.9	F1A—C1A—F2A	108.25 (19)
C24—C23—H23B	108.9	F3A—C1A—F2A	106.79 (18)
H23A—C23—H23B	107.7	F1A—C1A—S1A	110.29 (15)
C23—C24—H24A	109.5	F3A—C1A—S1A	112.07 (15)
C23—C24—H24B	109.5	F2A—C1A—S1A	111.55 (15)
			
N2—Fe1—N1—C1	−176.28 (16)	Fe1—N3—C11—C10	−9.3 (3)
N3—Fe1—N1—C1	100.3 (3)	C14—N3—C11—C12	−0.2 (2)
N4—Fe1—N1—C1	17.74 (16)	Fe1—N3—C11—C12	172.20 (13)
O1A—Fe1—N1—C1	−78.82 (16)	C10—C11—C12—C13	−177.9 (2)
N2—Fe1—N1—C4	−8.70 (16)	N3—C11—C12—C13	0.5 (2)
N3—Fe1—N1—C4	−92.1 (3)	C10—C11—C12—C29	2.8 (3)
N4—Fe1—N1—C4	−174.68 (16)	N3—C11—C12—C29	−178.71 (18)
O1A—Fe1—N1—C4	88.77 (16)	C11—C12—C13—C14	−0.6 (2)
N1—Fe1—N2—C6	9.46 (17)	C29—C12—C13—C14	178.6 (2)
N3—Fe1—N2—C6	176.80 (17)	C11—C12—C13—C31	179.8 (2)
N4—Fe1—N2—C6	92.8 (3)	C29—C12—C13—C31	−0.9 (4)
O1A—Fe1—N2—C6	−88.49 (17)	C11—N3—C14—C15	179.5 (2)
N1—Fe1—N2—C9	176.31 (17)	Fe1—N3—C14—C15	7.1 (3)
N3—Fe1—N2—C9	−16.36 (17)	C11—N3—C14—C13	−0.2 (2)
N4—Fe1—N2—C9	−100.3 (3)	Fe1—N3—C14—C13	−172.55 (14)
O1A—Fe1—N2—C9	78.35 (17)	C12—C13—C14—C15	−179.1 (2)
N1—Fe1—N3—C14	−90.9 (3)	C31—C13—C14—C15	0.4 (4)
N2—Fe1—N3—C14	−174.27 (17)	C12—C13—C14—N3	0.5 (2)
N4—Fe1—N3—C14	−8.30 (17)	C31—C13—C14—N3	−179.9 (2)
O1A—Fe1—N3—C14	88.29 (17)	N3—C14—C15—C16	−1.7 (4)
N1—Fe1—N3—C11	98.4 (3)	C13—C14—C15—C16	178.0 (2)
N2—Fe1—N3—C11	15.00 (16)	C14—C15—C16—N4	0.6 (3)
N4—Fe1—N3—C11	−179.04 (16)	C14—C15—C16—C17	−177.2 (2)
O1A—Fe1—N3—C11	−82.44 (16)	C19—N4—C16—C15	−176.66 (19)
N1—Fe1—N4—C19	−15.57 (16)	Fe1—N4—C16—C15	−5.1 (3)
N2—Fe1—N4—C19	−99.0 (3)	C19—N4—C16—C17	1.4 (2)
N3—Fe1—N4—C19	177.07 (16)	Fe1—N4—C16—C17	172.91 (13)
O1A—Fe1—N4—C19	82.34 (16)	C15—C16—C17—C18	176.11 (19)
N1—Fe1—N4—C16	174.72 (17)	N4—C16—C17—C18	−1.9 (2)
N2—Fe1—N4—C16	91.3 (3)	C15—C16—C17—C33	−6.5 (3)
N3—Fe1—N4—C16	7.36 (16)	N4—C16—C17—C33	175.49 (17)
O1A—Fe1—N4—C16	−87.37 (16)	C16—C17—C18—C19	1.6 (2)
C4—N1—C1—C20	177.06 (18)	C33—C17—C18—C19	−175.66 (19)
Fe1—N1—C1—C20	−13.1 (3)	C16—C17—C18—C35	−178.72 (19)
C4—N1—C1—C2	−1.3 (2)	C33—C17—C18—C35	4.0 (3)
Fe1—N1—C1—C2	168.54 (13)	C16—N4—C19—C20	179.99 (18)
C20—C1—C2—C3	−176.16 (19)	Fe1—N4—C19—C20	8.4 (3)
N1—C1—C2—C3	2.2 (2)	C16—N4—C19—C18	−0.4 (2)
C20—C1—C2—C21	4.0 (3)	Fe1—N4—C19—C18	−171.93 (13)
N1—C1—C2—C21	−177.66 (18)	C17—C18—C19—C20	178.81 (19)
C1—C2—C3—C4	−2.1 (2)	C35—C18—C19—C20	−0.9 (3)
C21—C2—C3—C4	177.77 (19)	C17—C18—C19—N4	−0.8 (2)
C1—C2—C3—C23	179.52 (19)	C35—C18—C19—N4	179.49 (17)
C21—C2—C3—C23	−0.6 (3)	N1—C1—C20—C19	−1.2 (3)
C1—N1—C4—C5	177.24 (19)	C2—C1—C20—C19	176.93 (19)
Fe1—N1—C4—C5	7.5 (3)	N4—C19—C20—C1	3.7 (3)
C1—N1—C4—C3	0.0 (2)	C18—C19—C20—C1	−175.94 (19)
Fe1—N1—C4—C3	−169.74 (13)	C3—C2—C21—C22	91.9 (3)
C2—C3—C4—C5	−175.89 (19)	C1—C2—C21—C22	−88.3 (3)
C23—C3—C4—C5	2.6 (3)	C2—C3—C23—C24	90.5 (3)
C2—C3—C4—N1	1.4 (2)	C4—C3—C23—C24	−87.6 (3)
C23—C3—C4—N1	179.85 (18)	C8—C7—C25—C26	−93.2 (3)
N1—C4—C5—C6	−3.6 (3)	C6—C7—C25—C26	79.7 (3)
C3—C4—C5—C6	173.3 (2)	C7—C8—C27—C28	96.3 (3)
C4—C5—C6—N2	4.5 (3)	C9—C8—C27—C28	−79.0 (3)
C4—C5—C6—C7	−173.1 (2)	C13—C12—C29—C30	−93.5 (3)
C9—N2—C6—C5	−178.31 (19)	C11—C12—C29—C30	85.6 (2)
Fe1—N2—C6—C5	−9.2 (3)	C12—C13—C31—C32	92.8 (4)
C9—N2—C6—C7	−0.5 (2)	C14—C13—C31—C32	−86.7 (3)
Fe1—N2—C6—C7	168.65 (14)	C12—C13—C31—C32'	−108.5 (3)
C5—C6—C7—C8	177.9 (2)	C14—C13—C31—C32'	72.0 (4)
N2—C6—C7—C8	0.0 (2)	C18—C17—C33—C34	101.2 (2)
C5—C6—C7—C25	3.7 (3)	C16—C17—C33—C34	−75.7 (2)
N2—C6—C7—C25	−174.2 (2)	C17—C18—C35—C36	−97.2 (2)
C6—C7—C8—C9	0.5 (2)	C19—C18—C35—C36	82.4 (3)
C25—C7—C8—C9	174.3 (2)	O3A—S1A—O1A—Fe1	−69.33 (13)
C6—C7—C8—C27	−175.5 (2)	O2A—S1A—O1A—Fe1	69.37 (13)
C25—C7—C8—C27	−1.7 (4)	C1A—S1A—O1A—Fe1	179.23 (11)
C6—N2—C9—C10	−178.53 (19)	N1—Fe1—O1A—S1A	−2.03 (12)
Fe1—N2—C9—C10	12.3 (3)	N2—Fe1—O1A—S1A	88.37 (12)
C6—N2—C9—C8	0.8 (2)	N3—Fe1—O1A—S1A	178.16 (11)
Fe1—N2—C9—C8	−168.44 (14)	N4—Fe1—O1A—S1A	−91.95 (12)
C7—C8—C9—C10	178.5 (2)	O3A—S1A—C1A—F1A	61.84 (19)
C27—C8—C9—C10	−5.3 (3)	O2A—S1A—C1A—F1A	−62.50 (18)
C7—C8—C9—N2	−0.8 (2)	O1A—S1A—C1A—F1A	−179.77 (16)
C27—C8—C9—N2	175.4 (2)	O3A—S1A—C1A—F3A	−178.18 (15)
N2—C9—C10—C11	0.1 (3)	O2A—S1A—C1A—F3A	57.48 (17)
C8—C9—C10—C11	−179.1 (2)	O1A—S1A—C1A—F3A	−59.79 (17)
C9—C10—C11—N3	−1.7 (3)	O3A—S1A—C1A—F2A	−58.49 (18)
C9—C10—C11—C12	176.6 (2)	O2A—S1A—C1A—F2A	177.17 (15)
C14—N3—C11—C10	178.29 (19)	O1A—S1A—C1A—F2A	59.90 (17)
